# Breast MRI and tumour biology predict axillary lymph node response to neoadjuvant chemotherapy for breast cancer

**DOI:** 10.1186/s40644-019-0279-4

**Published:** 2019-12-26

**Authors:** Samia Al-Hattali, Sarah J. Vinnicombe, Nazleen Muhammad Gowdh, Andrew Evans, Sharon Armstrong, Douglas Adamson, Colin A. Purdie, E. Jane Macaskill

**Affiliations:** 1 Department of Breast Surgery, Ninewells Hospital and Medical School, Dundee, DD1 9SY UK; 2 Thirlestaine Breast Centre, Cheltenham, UK; 3 Ninewells Hospital and Medical School, Dundee, UK; 4 Department of Breast Radiology, Ninewells Hospital and Medical School, Dundee, UK; 5 Department of Clinical Oncology, Ninewells Hospital and Medical School, Dundee, UK; 6 Department of Breast Pathology, Ninewells Hospital and Medical School, Dundee, UK

**Keywords:** Neoadjuvant chemotherapy, Axilla lymph node, Breast cancer, Magnetic resonance imaging, Sentinel node biopsy

## Abstract

**Background:**

In patients who have had axillary nodal metastasis diagnosed prior to neoadjuvant chemotherapy for breast cancer, there is little consensus on how to manage the axilla subsequently. The aim of this study was to explore whether a combination of breast magnetic resonance imaging (MRI) assessed response and primary tumour pathology factors could identify a subset of patients that might be spared axillary node clearance.

**Methods:**

A retrospective data analysis was performed of patients with core biopsy-proven axillary nodal metastasis prior to commencement of neoadjuvant chemotherapy (NAC) who had subsequent axillary node clearance (ANC) at definitive breast surgery. Breast tumour and axillary response at MRI before, during and on completion of NAC, core biopsy tumour grade, tumour type and immunophenotype were correlated with pathological response in the breast and the number of metastatic nodes in the ANC specimens.

**Results:**

Of 87 consecutive patients with MRI at baseline, interim and after neoadjuvant chemotherapy who underwent ANC at time of breast surgery, 33 (38%) had no residual macrometastatic axillary disease, 28 (32%) had 1–2 metastatic nodes and 26 (30%) had more than 2 metastatic nodes. Factors that predicted axillary nodal complete response were MRI complete response in the breast (*p* < 0.0001), HER2 positivity (*p* = 0.02) and non-lobular tumour type (*p* = 0.015).

**Conclusion:**

MRI assessment of breast tumour response to NAC and core biopsy factors are predictive of response in axillary nodes, and can be used to guide decision making regarding appropriate axillary surgery.

## Introduction

In the past two decades, sentinel node biopsy (SNB) has become standard practice for surgically staging the axilla in patients having primary surgery for clinically node-negative breast cancers, replacing the more morbid procedure of axillary node clearance (ANC). With increasing use of neoadjuvant chemotherapy (NAC) to downsize breast tumours to achieve breast conservation, there is little consensus on how to manage the axilla, with varying practices including SNB before or after chemotherapy and ANC [1]. In many units, ANC has remained the definitive axillary procedure after NAC if pre-treatment axillary ultrasound (AUS) and core biopsy confirmed nodal positivity. However, NAC has been demonstrated to eradicate nodal metastasis in up to 40% of patients with human epidermal growth factor receptor 2 (HER2) negative tumours, and up to 75% of HER2 positive patients treated with trastuzumab [2–5].

The feasibility and accuracy of SNB post-NAC in clinically node-negative patients has been established, with false-negative rates (FNR) of 8–11% [6–12]. A recent meta-analysis of the accuracy and reliability of sentinel lymph node biopsy after NAC in patients with initial biopsy-proven node-positive breast cancer demonstrated a false-negative rate with use of dual techniques of 11% compared with 19% with single mapping [13]. It also confirmed findings from previous studies showing that a higher number of nodes removed improved accuracy (FNR 20% when one node was removed, 12% with two nodes removed and 4% with removal of three or more nodes) [14–17].

It is known that the response within axillary nodal metastases correlates with that seen in the breast tumour [14, 18]. However, it would be advantageous to determine pre-operatively which patients have had a sufficiently good response to NAC in the axilla to allow potential downstaging of axillary surgery and avoidance of the morbidity of an unnecessary ANC.

The accuracy of MRI in predicting the presence of residual breast disease after NAC has been demonstrated in several studies and systematic reviews [19–21], with high negative predictive values (NPV) for pathological Complete Response (pCR), particularly in HER2 positive and triple negative disease [22]. What is not clear is the extent to which MRI reflects residual axillary nodal disease and response in the axilla in the context of neoadjuvant chemotherapy. Indeed, a systematic review of non-invasive nodal restaging in node-positive patients after NAC from 2015 found extreme heterogeneity precluding pooled analysis, and highly variable reported positive predictive values for axillary pathological complete response. It concluded that there were no accurate non-invasive restaging techniques [23]. Thus, this remains an area of unmet clinical need.

The aim of this study was to attempt to identify a subset of patients defined by MRI assessed response and primary tumour pathology factors who might be spared ANC.

## Methods

This was a retrospective audit of data from a consecutive series of patients with a pathological diagnosis of breast cancer and proven nodal metastasis who received NAC within a single centre between September 2010 to June 2015. Only patients who had undergone ultrasound-guided core biopsy of ultrasonographically abnormal nodes (cortical thickness > 2.3 mm) with proven axillary node metastasis prior to starting chemotherapy were included. After completion of NAC, all patients underwent axillary node clearance (ANC) as per the standard local policy, regardless of the response. Local ethical approval was obtained for collection, analysis and presentation of data.

Patients were scheduled for breast MRI to assess the primary tumour and axilla before (pre-treatment), after 3 cycles (interim) and at the end of (post) chemotherapy. T2 weighted sequences and standard semi-dynamic contrast-enhanced MRI was performed with a fat-suppressed T1 weighted spoiled gradient echo sequence, time 45–50 s per acquisition, repeated out to 8 min.

Final MRI response was assessed according to modified Response Evaluation Criteria in Solid Tumours (RECIST) 1.1, and categorised as complete response (MRI CR), partial response (MRI PR), stable disease (MRI SD), or progressive disease (MRI PD) [24], based on the presence and size of enhancing masses and non-mass enhancement. No enhancement in the tumour bed or in a residual mass, or no enhancement above background parenchymal enhancement at any phase of contrast-enhanced imaging, were denoted MRI CR; an MRI PR occurred when a residual mass or area of non-mass enhancement had reduced by more than 30% in maximum diameter. MRI results were derived from the clinical report, with review of axillary findings on MRI re-reported by an experienced breast radiologist (SJV) blinded to the pathological outcome.

Axillary nodes were assessed on MRI as normal, abnormal or borderline on each of the three MRI scans according to nodal morphology and size on T2 weighted and contrast enhanced T1 weighted sequences. Normal lymph nodes were those with uniform cortical thicknesses no more than 2.5 mm. Borderline nodes were those with mild cortical thickening more than 2.5 mm, either uniform or eccentric, and abnormal nodes were those with clearly abnormal size and morphology. At ultrasound, cortical thickness more than 2.3 mm was described as abnormal. Where ultrasound nodal response had not been recorded, available images were reviewed and assessed retrospectively by a radiologist (NMG) blinded to the MRI response to treatment.

Pathological response in the primary breast tumour was classified as: [25]

1. Pathologic complete Response (pCR), no residual invasive disease present;
2. Near complete Response, the residual invasive disease has a percentage reduction in cellularity of > = 90%;
3. Partial Response, reduction in cellularity of > 50% and < 90%;
4. Minimal Response, reduction in cellularity 1–50%;
5. No pathological response, 0% reduction in cellularity.

Axillary lymph nodes were considered negative on pathology if no macrometastasis (foci > 2.0 mm) was identified on standard haematoxylin and eosin staining. The presence of scarring, micrometastasis (Mic) (foci 0.2–2.0 mm) or isolated tumour cells (ITC) (foci < 0.2 mm or < 200 cancer cells in one section) was recorded and analysed.

The response of the breast tumour on MRI, and of the axillary nodes on MRI and ultrasound, together with core biopsy tumour grade, tumour type and immunophenotype were correlated with the final pathology response in the breast and the number of abnormal nodes in the nodal clearance specimens.

The data were plotted in contingency tables and the significance tests used were Chi squared, Fisher exact, and Pearson correlation using *vassarstats.com*. Sensitivity, specificity and univariate analysis was performed using *SPSS* v 22.0.

## Results

Of 176 patients treated with NAC during the study period, 117 patients had core biopsy-confirmed axillary nodal metastasis prior to treatment. Eighty-seven patients had available MRI data for analysis from before, during and after NAC and thus formed the study cohort. This number included two patients who were planned to receive 4 cycles only of NAC, and had MRI before and after 4 cycles (called interim for analysis purposes), and 4 patients who received 6 cycles and had only pre-and post treatment MRI scans.

The median age was 50 years (range 24–79). Baseline characteristics are shown in Table 1. In eight patients, low-volume distant metastatic disease was identified after commencing NAC, which was continued and patients underwent surgery as planned. Sixty-eight patients (74.2%) received a combination of anthracycline-based chemotherapy (5-fluorouracil, epirubicin and cyclophosphamide) and taxane, and 34 patients (39.1%) had trastuzumab in combination with chemotherapy (Table 1). Median time to surgery was 6 weeks (4–12 weeks) after the last cycle of chemotherapy. In one patient surgery was delayed for 12 weeks resulting from investigation of a suspicious liver lesion later confirmed as benign. Fifty-eight patients (66.7%) underwent mastectomy and 29 (33.3%) had breast conserving surgery. The median number of lymph nodes retrieved at axillary clearance was 18 (7–34). The baseline pathological features are detailed in Table 1.

**Table 1:** Baseline tumour pathology and treatment characteristics

	Total patients *n* = 87 n (%)				
cT stage	T1	T2	T3	T4	
	1 (1.1%)	53 (60.9%)	18 (20.7%)	15 (17.2%)	
cN stage	N1	N2	N3		
	76 (86%)	7 (9%)	4 (5%)		
Type of chemotherapy	Anthracycline only	Taxane only	Combination		
	14 (16.1%)	5 (5.7%)	68 (78.2%)		
Trastuzumab	Trastuzumab				
	34 (39.1%)				
Type of surgery	Breast conservation		Mastectomy		
	29 (33.3%)		58 (66.7%)		
Tumour grade	G1	G2	G3		
	1 (1.1%)	23 (26.4%)	63 (72.4%)		
Tumour type	Invasive ductal	Invasive lobular	Other		
	79 (90.8%)	4 (4.6%)	4 (4.6%)		
Immunophenotype	ER +/HER2 +	ER +/HER2	ER −/HER2	ER −/HER2 –	
	26 (29.9%)	30 (34.5%)	8 (9.2%)	23 (26.4%)	
Pathological response of tumour	Complete response	Near complete	Partial response	Minimal response	No response
	15 (17.2%)	21 (24.1%)	41 (47.1%)	2 (2.3%)	8 (9.2%)
Pathological assessment of nodes	No residual metastasis	ITC/ Mic only	1–2 nodes with macrometasis	More than 2 nodes with metastasis	
	23 (26.4%)	10 (11.5%)	28 (32.3%)	26 (29.9%)	

### Pathological assessment of breast tumour and axillary nodal response

In the breast, pCR was reported in 15 patients (17.2%), near CR in 21 patients (24.1%), PR in 41 patients (47.1%), minimal response in 2 patients (2.3%) and no response in 8 patients (9.2%) (Table 1).

Axillary node clearance pathological assessment revealed no residual lymph node macrometastasis in 33 patients (37.9%), including 10 patients with residual ITC or Mic identified. Twenty-eight patients (32.2%) had one or two lymph nodes with macrometastasis, whereas 26 patients (29.9%) had more than 2 lymph nodes with residual macrometastasis (Tables 1 & 2). Twenty-eight patients had ITC or Mic disease, of whom 10 had only ITC/Mic, 7 had also 1 or 2 nodes with macrometastasis, and 11 had more than 2 nodes with macrometastasis. In approximately half of the patients there was evidence of nodal scarring in response to treatment in one or more lymph nodes. Data were analysed including ITC and Mic disease as a separate group, with no statistical differences, and data shown in Table 2 include ITC and Mic disease categorised as no residual macrometastatic disease.

**Table 2:** Patient and tumour factors and association with level of axillary nodal burden on pathology

	No residual macrometastatic nodes n (%)	1–2 residual metastatic nodes n (%)	More than 2 residual metastatic nodes n (%)	*p*-value
Total	33 (37.9%)	28 (32.2%)	26 (29.9%)	
Patient median age (years)	50.0	48.5	53.0	0.319
Trastuzumab given				
yes	18 (53%)	8(23.5%)	8 (23.5%)	0.068
no	15 (28%)	20 (38%)	18 (34%)	
Type of chemo				
FEC	2 (14%)	7 (50%)	5 (36%)	
Taxane	2 (40%)	2 (40%)	1 (20%)	0.325
both	29 (43%)	19 (28%)	20 (29%)	
Tumour type				
Ductal	30 (38%)	27 (34%)	22 (28%)	0.015
Lobular	0 (0%)	0 (0%)	4 (100%)	
Other	3 (75%)	1 (25%)	0 (0%)	
Tumour grade				
1	0 (0%)	1 (100%)	0 (0%)	0.080
2	6 (26%)	12 (52%)	5 (22%)	
3	27 (43%)	15 (24%)	21 (33%)	
T staging				
T1	1 (100%)	0 (0%)	0 (0%)	0.188
T2	21 (40%)	21 (40%)	11 (20%)	
T3	7 (39%)	3 (17%)	8 (44%)	
T4	4 (27%)	4 (27%)	7 (46%)	
Immunophenotype				
ER + ve/HER2 + ve	13 (50%)	8 (31%)	5 (19%)	
ER + ve/HER2 -ve	6 (20%)	15 (50%)	9 (30%)	0.044
ER -ve/HER2 + ve	5 (63%)	0 (0%)	3 (37%)	
ER -ve/HER2 –ve	9 (39%)	5 (22%)	9 (39%)	

There was a statistically significant correlation between breast tumour and axillary response as assessed by pathology: 86.7% (13 of 15) of those with tumour pCR, and 52.4% (11 of 21) of those who achieved near complete response in the primary breast tumour had no residual axillary disease respectively. In comparison, only 19.5% (8 of 41) of those with partial response, and 10% (1 of 10) of those with no or minimal response in the breast tumour proved to have no residual axillary disease respectively (*r* = 0.495; *p* < 0.00001).

### Core biopsy factors and MRI assessment of breast and axillary nodal response

Results of analysis of all treatment and tumour variables and association with nodal response to treatment are shown in Table 2. Patients with HER2 positive disease (including both oestrogen receptor (ER) positive and ER negative disease) had a significantly higher rate of nodal complete response (*p* = 0.04). Although only small numbers of patients with lobular tumours were included, all had high volume residual nodal disease (4 of 4 patients, *p* = 0.015) (Table 2).

MRI response in the breast correlated significantly with pathological response (*r* = 0.690; *p* < 0.000001).

**Fig. 1:**
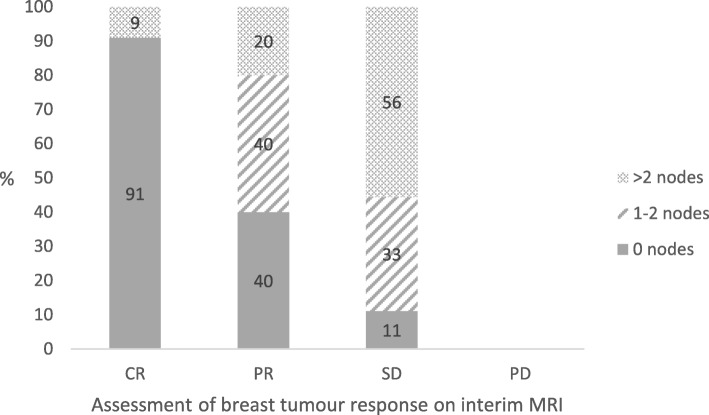
Demonstrates the association of tumour response as predicted on interim-treatment breast MRI and the pathological findings in axillary nodes *r* = 0.50; *p* < 0.0001. There were no patients with tumours showing progressive disease at interim MRI. Key: No residual macrometastasis in axillary nodes =0; residual macrometastasis in 1 or 2 nodes =1–2; residual macrometastasis in more than 2 nodes = > 2

Both interim and end-of-treatment MRI assessment of response in the breast correlated with nodal burden at ANC (*p* < 0.0001 for both) (Table 3) (Fig. 1). Only one of 10 patients (10%) with an MR CR at interim MRI had positive lymph nodes after treatment. In contrast, all those with stable disease on interim MRI had residual macroscopic nodal disease (no patients had progressive disease at interim MRI) (Table 3) (Fig. 1). The overall node-negative conversion rate was 40% for the MRI PR group, with a further 40% having 1–2 residual nodes, and 20% with more than 2 positive nodes (Fig. 1). Core biopsy tumour grade did not correlate with nodal response.

**Table 3:** Presurgical imaging modalities and association with level of axillary nodal burden on pathology

	No residual macrometastatic nodes n (%)	1–2 residual metastatic nodes n (%)	More than 2 residual metastatic nodes n (%)	*p*-value
Total	33 (38%)	28 (32%)	26 (30%)	
MRI breast tumour phenotype:				
Mass	21 (41%)	20 (39%)	10 (20%)	
Non-mass enhancement	4 (33%)	4 (33%)	4 (33%)	0.091
Both	8 (33%)	4 (17%)	12 (50%)	
MRI breast interim chemo:				
Complete response	10 (90%)	0	1 (10%)	
Partial response	18 (40%)	18 (40%)	9 (20%)	< 0.0001
Stable disease	3 (11%)	9 (33%)	15 (56%)	
Progressive disease	0 (0%)	0 (0%)	0 (0%)	
MRI breast post chemo:				
Complete response	25 (64%)	9 (23%)	5 (13%)	< 0.0001
Partial response	6 (15%)	16 (40%)	18 (45%)	
Stable disease	2 (40%)	2 (40%)	1 (20%)	
Progressive disease	0 (0%)	1 (100%)	0 (0%)	
MRI axilla post chemo:				
Normal	21 (46%)	13 (28%)	12 (26%)	0.087
Borderline	7 (39%)	7 (39%)	4 (22%)	
Abnormal (partial response)	5 (31%)	3 (19%)	8 (50%)	
Abnormal	0 (0%)	5 (71%)	2 (29%)

### Axillary imaging to predict response in axillary nodes

**Fig. 2:**
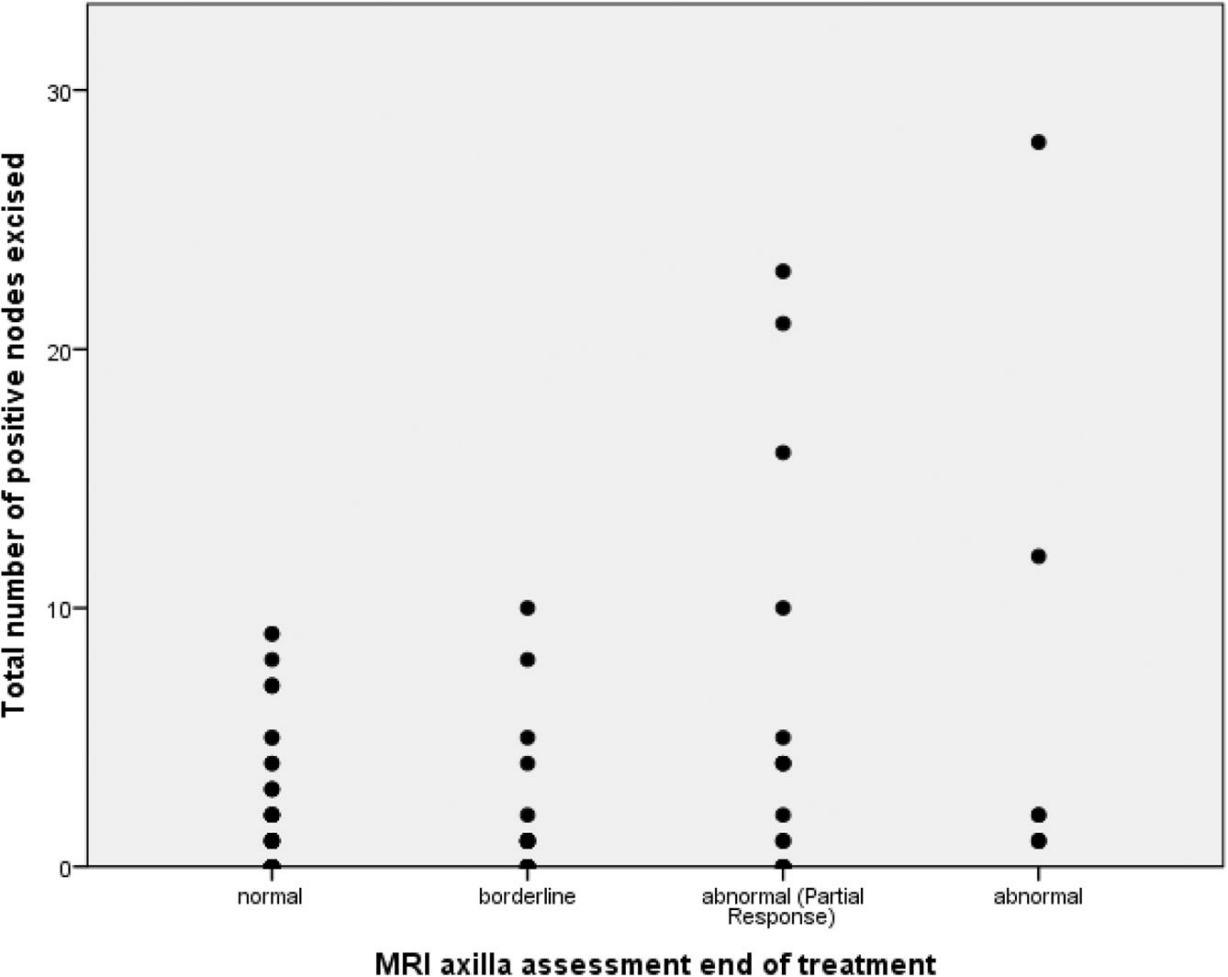
demonstrates the reported axillary response from MRI assessment post-treatment and the number of positive nodes from the axillary node clearance. There was no statistical correlation

The axilla was assessed on MRI in 87 patients, and on USS in only 48. There was no correlation with imaging assessment of the axilla at interim or end-of-treatment and nodal positivity rates, with high false-positive and false-negative rates for those with data available for axillary USS and for MRI (Table 3; Fig. 2) In the subgroup of patients with MRI-reported abnormal nodes prior to chemotherapy, and normal axillary MRI post-treatment (*n* = 37), there were no abnormal nodes on surgery in 18 patients (48.6%), 1–2 nodes in 9 (24.3%), and more than 2 residual positive nodes in 10 (27.0%). There was therefore a false-negative rate of MRI axillary assessment of 27% for those with higher nodal burden of residual disease. Of those with MRI reported abnormal axillary nodes after NAC, including nodes that remained abnormal but showed partial response to treatment, 5 of 23 (21.7%) had no residual axillary nodal disease at surgery, while 8 (34.8%) had 1–2 nodes positive, and 10 (43.5%) had more than 2 positive nodes, thus a false-positive rate of 21.7%.

## Discussion

We have shown in this series that 38% of patients will have no residual macrometastatic axillary disease after NAC, which is similar to that of NSABP B-18 and American College of Surgeons Oncology Group trial (ACOSOG Z1071) with 37 and 41% respectively [26], and as such these patients could be spared ANC, with consideration of axillary radiotherapy.

In the ACOSOG Z1071 trial the highest pCR rates in both the breast and axilla were seen in HER2 positive cancers at 45.4%, followed by triple negative cancers at 38.2%, whereas the pCR rate was only 11.4% in the hormone-receptor positive category [14] The overall pCR rate of 17.2% in our study was in accordance with the expected rates taking in consideration that approximately two thirds of our study population were hormone-receptor positive [14]. Our data show a better response in nodal disease in HER2 positive tumours, with no response in axillary nodes in the small number of lobular cancers included.

The response in the breast at interim and end-of-treatment MRI was found to correlate well with the pathological response rates, as in previous studies [19–22]. This provides an opportunity to assess response in the breast and also, as shown in our data, to aid decision-making regarding axillary surgery. Amongst all breast imaging modalities, contrast-enhanced (DCE) MRI has the best accuracy and positive predictive value (PPV) for the presence of residual breast disease. Furthermore, it has the best NPV in predicting complete pathological response (pCR) [19].

Our study was retrospective, utilising diagnostic MRI images as performed in many breast centres, and as such the metric chosen for analysis is one that can be employed in any breast centre. We suggest that our results are more likely to be generalisable, since they are independent of the postprocessing platform utilised. Diffusion weighted imaging, although sensitive particularly in identification of pCR, has the issue of a current lack of standardisation across centres.

Both interim and end-of-treatment MRI were performed in our patient group. It is plausible patients whose tumours respond well at MRI after only 3–4 cycles are very likely to have good nodal response, but there was still a sizeable minority of patients with interim MRI PR who went on to have MRI CR on completion of NAC (around 30%). Our data therefore suggest that assessment of breast response on MRI should be performed towards the end of NAC with sufficient time to allow for surgical planning, but that a series of 3 MRIs during treatment may not be necessary.

Stratification of tumour response on breast MRI has been shown in our series to be indicative of axillary nodal response. In accordance with the results of a systematic review, we did not find that MRI assessment of the axillary nodes was helpful in this context, as there were high false-positive and false-negative rates [23]. This is despite the fact that the breast tumour response was evident at the time of reporting to the radiologist, and one might expect some bias towards favourable assessment of the axilla if breast tumour response is good, but our data do not support this. Thus use of MRI to assess the axilla does not aid decision making on axillary clearance in our series. Importantly, when the axilla normalised at MRI, over 54% of patients proved to have one or more residual macrometastases at ANC.

Axillary USS (AUS) was not performed after treatment in a number of patients in this series, as during the study period it did not influence the outcomes for the patient because of the local policy recommending ANC if nodal disease had been proven prior to NAC. For those in whom AUS results were available it did not appear to be useful in addition to breast MRI response. AUS has previously been shown to surpass other modalities in axillary lymph node assessment [19], although there has been a wide variability in the reported sensitivity (27–94%) and specificity (53–98%) [27, 28]. In order to ascertain any additional benefit from AUS assessment of axillary nodes after ANC, we are now routinely performing this at the end of NAC.

This study has some limitations, with relatively small numbers of patients from a single institution. Nonetheless the results suggest that the findings are worthy of consideration in a larger cohort of patients, as the importance of breast MRI in prediction of axillary response after NAC has not been well studied thus far. Our data indicate that, taken together with tumour specific factors, breast MRI may be able to guide surgical decision making for the axilla in the increasing number of breast cancer patients treated with NAC.

## Conclusions

Contrast-enhanced MRI assessed response in the primary breast tumour is very useful for predicting response in the axilla, but axillary node assessment of response by DCE-MRI is less accurate. An MRI complete response in the breast was the only imaging factor we found to predict for axillary response. If one considers that axillary radiotherapy or perhaps no further local treatment may be an acceptable alternative to ANC in patients with no macrometastatic axillary disease after NAC, then a third of patients in this series could have been spared ANC. From analysis of these data, our local policy has been changed to incorporate MRI breast response, immunophenotype tumour type including HER2 positive and non-lobular type tumours, and AUS where available to identify patients who now undergo SNB rather than ANC, and we are auditing this change of practice.

## Data Availability

The datasets generated during and/or analyses during the current study are not publicly available due to patient confidentiality but are available anonymised from the corresponding author on reasonable request.

## Abbreviations

ACOSOG: American College of Surgeons Oncology Group
ANC: Axillary node clearance
AUS: Axillary ultrasound scan
DCE MRI: Dynamic contrast enhanced magnetic resonance imaging
ER: Oestrogen receptor
FNR: False negative rate
HER2: Human epidermal growth factor receptor 2
ITC: Isolated tumour cells
Mic: Micrometastasis
MRI- CR: Magnetic resonance imaging complete response
MRI- PD: Magnetic resonance imaging progressive disease
MRI- PR: Magnetic resonance imaging partial response
MRI- SD: Magnetic resonance imaging stable disease
MRI: Magnetic resonance imaging
NAC: Neoadjuvant chemotherapy
NPV: Negative predictive value
NSABP: National Surgical Adjuvant Breast Project
pCR: Pathological complete response
PPV: Positive predictive value
RECIST: Response Evaluation Criteria in Solid Tumours
ROC: Receiver operating characteristic
SNB: Sentinel node biopsy
